# Taking the LEAP: study protocol for a randomized, multicentre, naturalistic, efficacy trial of the compuLsive Exercise Activity theraPy (LEAP) - a cognitive behavioral program specifically targeting compulsive exercise in patients with eating disorders

**DOI:** 10.1186/s12888-021-03356-2

**Published:** 2021-07-23

**Authors:** Elin Monell, Caroline Meyer, Agnieszka Szwajda, Emma Forsén Mantilla

**Affiliations:** 1 Department of Medical Biostatistics and Epidemiology, Karolinska Institutet, Stockholm, Sweden; 2 Centre for Psychiatry Research, Department of Clinical Neuroscience, Karolinska Institutet & Stockholm Health Care Services, Stockholm, Sweden; 3 WMG and Warwick Medical School, University of Warwick, Coventry, UK; 4 Warwickshire NHS Partnership Trust, Coventry, UK

**Keywords:** Compulsive exercise, Eating disorders, Treatment, Efficacy trial

## Abstract

**Background:**

About half of Swedish eating disorder patients report exercising compulsively and compulsive exercise (CE) is prevalent in all diagnoses and both genders. Yet there are no systematic treatments targeting CE in specialist care. This study aims to evaluate the effects of The CompuLsive Exercise Activity TheraPy (LEAP) - a promising group treatment targeting compulsive exercise, in Swedish eating disorder patients.

**Method:**

One hundred twenty-eight adult females and males suffering from anorexia nervosa, bulimia nervosa or other specified feeding and eating disorders (type 1, 2, or 4) with CE will be recruited via four specialist eating disorder treatment units. Participants will be randomized to receive treatment as usual (control group) or treatment as usual plus LEAP (intervention group). The groups will be assessed on key variables (e.g., BMI, eating disorder symptoms, exercise cognitions and behaviors) at three occasions: initially, after 3 months and after 6 months.

**Discussion:**

The project takes place in a clinical setting, including both male and female patients with different eating disorder diagnoses with CE, enabling a good indication of the efficacy of LEAP. If our results are positive, LEAP has the potential of benefiting about half of the eating disorder population, with remission and recovery hopefully improving as a result.

**Trial registration:**

The trial is registered with the ISRCTN registry (registration date 2020-03-25), trial ID: ISRCTN80711391.

## Background

Compulsive exercise (CE) is a key factor in eating disorder (ED) etiology, development, and maintenance [1, 2]. In adult patients, increased levels of CE are related to more severe psychopathology, increased suicidality, longer hospitalizations, increased relapse risk, and a more chronic course in anorexia nervosa (AN [1, 3–5];). Patients who continue or initiate CE during treatment have 1-year remission rates at roughly half of that among patients without CE or patients who discontinue CE during treatment [3]. In our nationwide study, CE was reported by about half of all ED patients [3]. Previous studies have found CE prevalence to be highest in AN [6], but in our sample patients with bulimia nervosa (BN) and patients with other specified feeding and eating disorders (OSFED) reported the highest prevalence of CE [3]. More importantly, CE was prevalent in all ED diagnoses and almost as common in both genders. Of note, a 2011 nationwide study on Swedish ED patients [7] found lower overall CE rates, but higher rates of binge-eating and purging, than we did. This suggests that CE is becoming more prevalent as a symptom, and/or that there might be gradual symptom-shifting among ED patients over the years.

To date, there is no “gold standard” treatment to tackle CE. However, ours and others’ results indicate a clear need to systematically address CE in ED treatment. The empirically based, targeted programme LEAP [8] aims to educate patients about CE and how it is maintained, challenge maladaptive beliefs and behaviors, and equip patients with strategies promoting healthy exercise. LEAP is founded on the same theoretical principles as the evidence-based ED treatment Cognitive Behavioral Therapy Enhanced (CBT-E [9];) and may be offered as a complement to CBT-E. LEAP is delivered in one initial individual session and eight group sessions. LEAP has been evaluated in two pilot studies and one RCT. In these studies, focusing adult patients with AN, LEAP reduced pathological exercise, ED psychopathology, general psychopathology, and length of hospitalization; it also improved attitudes and beliefs toward exercise (which in turn has proven important for outcome [10];), BMI, and quality of life [11–13]. These findings need to be confirmed at other sites and with other ED diagnostic groups. Of note, the patients in the RCT had suffered from AN (known as an illness notoriously difficult to treat) for an average of 6 years- a considerably long time, yet they made important progress. The effects of LEAP with other ED diagnostic groups and patients with shorter illness duration may therefore be even greater. To our knowledge, there are no existing systematic efforts targeting CE in Swedish specialist care.

## Method

### Objectives, design and setting

#### Objectives

The project aims to evaluate the efficacy of LEAP at reducing pathological exercise and improving ED pathology in Swedish patients diagnosed with AN, BN, or OSFED, in a naturalistic randomized controlled trial (RCT).

##### Primary research question

What are the treatment effects of LEAP on ED diagnosis, ED cognitions, CE behaviors and cognitions, BMI, emotion regulation and general psychopathology, after completion of the LEAP protocol.

##### Secondary research questions

Are there initial factors (e.g., BMI, emotion regulation, compulsivity) that predict a more favorable outcome for patients in the LEAP group? Is LEAP perceived as a feasible and acceptable treatment for patients?

#### Study design

The trial is a naturalistic two-armed parallel open-label superiority randomized efficacy trial, with recruitment from four Swedish specialist ED clinics. Eligible adult patients will be recruited via the clinics and randomized to treatment as usual (TAU) or TAU+LEAP, where LEAP will run in parallel with TAU. Key outcome variables in both arms are measured at initial assessment, after 3 months, and 6 months after initial assessment. Although most TAU+LEAP participants are expected complete the LEAP program prior to T2 assessment, a few may terminate LEAP prior to T3 (see Participant timeline).

#### Setting

The project is a collaboration between Karolinska Institute (KI) and four Swedish specialist ED treatment units: Eriksbergsgården in Örebro, Region Örebro; Ätstörningsenheten in Göteborg; Ätstörningsenheten in Uppsala; and Stockholms Centrum för Ätstörningar in Stockholm. Project management is done at KI while recruitment and the intervention take place at the units. These are medium to large units providing equivalent evidence-based care. The project related processes are equivalent at the units.

##### Therapists

LEAP-therapists are clinicians with vast experience working with patients with eating disorders and some experience of addressing CE in this population. Therapists at study start are licensed psychologists, physiotherapists, and licensed psychotherapists; other medical professions with ED experience could be included later due to potential staff changes. All therapists delivering the LEAP groups have received the same training to maximize adherence. In case of staff changes in LEAP therapists, any new therapist will receive equivalent training. All LEAP session will be audiotaped; two random sessions from each LEAP group will be externally assessed to monitor adherence and fidelity.

### Participants, intervention, and recruitment

#### Eligibility criteria

*Inclusion criteria:* male and female patients, ≥18 years, with AN, BN or OSFED (1. Atypical AN, 2. BN with low frequency and/or limited duration of symptoms, 4. purging disorder/compensatory behaviors after small food amounts), reporting CE at initial assessment (e.g., measured by EDE-Q version 6, item 18, see *Instruments*), and receiving outpatient care at the specialized ED treatment units. As more females than males seek treatment, there will be more females than males in the final sample.

*Exclusion criteria:* inability to communicate in Swedish, psychotic disorder, and/or BMI < 14.

#### Study consent

Subjects are first informed orally by project affiliated unit staff with extensive knowledge on the project, followed by oral or written information from the project manager (also the LEAP principal investigator; PI) or project coordinator (PC). Formal participant information and written study consent are managed and collected through the online data collection and participant management system BASS Core Facility (BASS; see *Data collection and management*) using a governmentally and financially approved secure electronic signature system (Bank-ID; https://www.bankid.com/en). Written participant information in BASS include details about the project, participation, potential risks and benefits of participation, usage of participant data, how to find study results, insurance and remuneration, and the voluntary basis of participation. Contact details to the PI, the KI Data Protection Officer and the Swedish Data Protection Authority are also provided. Patients consent to project participation, to medical records being collected and to have their data collected as described. There are no ancillary studies; all collected data are related to this project.

#### Interventions

The intervention group receive LEAP in addition to standard outpatient care (TAU) running in parallel, while the control group receive only TAU. Standard outpatient care includes a medical contact, psychoeducation about EDs, in most cases a CBT-based treatment via a personal therapeutic contact and/or group therapy, and meal-support. Everyone has a personalized treatment plan. There are no systematic interventions focusing on pathological exercise in TAU, which means that TAU+LEAP will clearly deviate from standard care.

##### Choice of comparators (choice of control interventions)

These patients receive standard outpatient care (i.e., TAU). TAU was chosen as comparator due to the naturalistic study design; TAU with varying content is what patients displaying CE usually receive within specialized ED treatment settings.

##### LEAP

LEAP is a semi-structured, problem-oriented group CBT, with psychoeducation specifically focusing on physical activity, behavioral experiments, and cognitive activities. The overarching aim for LEAP is to promote “healthy” exercise. Specific aims are to educate patients about the maintenance of CE, promote insight into factors affecting beliefs and behaviors toward exercise, introduce skills to help challenge maladaptive beliefs and behaviors, introduce adaptive emotion coping strategies, and prevent relapse. Each group contains 4–8 participants. The program consists of 1 individual session (Session 0) and 8 one-hour group sessions over four consecutive weeks. Sessions are preceded by homework tasks. Groups will be held at the treatment units. Two therapists at each treatment unit and three additional therapists from the research group (two acting as supervisors) have been trained in LEAP by the project coordinators and Prof. Caroline Meyer (CM) and clinical psychologist Tara Cousins (TC).

##### The Swedish LEAP manual (Table 1)

Since the publication of the original manual [8], several modifications have been made in both research and clinical settings (e.g., individual treatment, addition of individual session 0, reordering of sessions). The Swedish LEAP manual has reordered the sessions and included the individual session 0 held prior to the LEAP group. The Swedish manual has also, in consultation with the originator CM, replaced the session on physical movement at a low weight in AN (“Activity AN”) with a session focusing perfectionism and self-compassion as this was considered more suitable for the target group (i.e., outpatients with mixed EDs). See Table 1 for a session overview of the Swedish LEAP manual.

**Table 1:** LEAP session overview

Session	Name	Content
Session 0	Individual LEAP Exercise Profile	Initial information and introduction of the CBT-model of the maintenance of compulsive exercise. Completion of the LEAP Exercise Profile and individual maintenance formulation.
Session 1	Orientation	Group introduction, presentation of central concepts, the CBT-model, and the Exercise Profiles. Introduction of the behavioral experiment, self-monitoring task, and homework.
Session 2	Healthy and Unhealthy Exercise	Review differences between healthy and unhealthy exercise behaviors and attitudes. Connect to the CBT-model and Exercise Profiles. Introduction of the cognitive restructuring technique.
Session 3	Myths and Facts	Highlight the difference between myths and facts, and how myths (false beliefs, assumptions) can maintain unhealthy attitudes and behaviors toward exercise.
Session 4	Compulsive Exercise and Eating Disorders	Presentation of the relationship between compulsive exercise and eating disorders, and their respective maintaining factors. Introduction of the problem solving and guided discovery techniques.
Session 5	Psychological Dependence on Mood Regulation and ‘Exercise Addiction’	Highlight the role of exercise in regulation mood and emotions, and the risk of psychological dependence (‘addicion’) to exercise. Introduction of alternative strategies to manage emotions.
Session 6	Behavioural Rigidity	Explore how strict rules and unrealistic standards results in rigid, compulsive behaviors, both in relation to exercise and other life areas. Introduction of the cost analysis technique.
Session 7	Perfectionism and Exercise	Highlight the relationship between perfectionism, self-criticism and compulsive exercise. Introduction of acceptance and self-compassion as alternative approaches towards performance.
Session 8	Initiating and Maintaining Factors	Explore differences in reasons for starting and continuing to exercise. Summarizing the LEAP intervention, techniques, remaining challenges and relapse prevention.

##### Modifications and discontinuation

LEAP will not be modified for individual participants. There are no formal criteria for intervention discontinuation; LEAP-therapists discuss potential adverse events for individual participants (e.g., severe psychiatric deterioration) with the PI who then formally decides on possible intervention discontinuation. No additional assessments are required in case of treatment discontinuation or study withdrawal.

#### Adherence

The LEAP intervention begins with an individual session where the intervention is individually conceptualized, assumed to increase personal relevance and motivation. All group sessions begin with an opportunity to raise questions and homework is always followed up. Participants are continually reminded of the importance of actively participating in the session and the importance of completing homework tasks, self-monitoring and behavioral experiments. Potential obstacles are examined to improve adherence.

#### Concomitant care

All patients receive individually personalized TAU. CE will not receive systematic attention since this is not standard practice. However, the attention given to exercise as a symptom may vary between therapists. Thus, to control for potential deviations from standard practice in this regard, both study and control participants will answer questions about treatment content at the last assessment point, that is, questions about the content of their treatment including how much attention has been given to pathological exercise, if so, in which ways. Additionally, LEAP therapists are encouraged not to have LEAP participants (neither controls nor study) in individual treatment. However, due to smaller unit size for three of four units, there might be violations to this guideline. If so, LEAP therapists are instructed to have equal numbers of intervention and control patients in individual treatment.

#### Provisions for post-trial care

Most participants are expected to remain within their TAU after project participation, however, no ancillary or post-trial care is expected due to project participation. All participants are covered by the KI insurance and a patient injury insurance (‘Patientskadeförsäkring’), covering compensation for those suffering potential harm from trial participation.

#### Participant timeline (Fig. 1)

Participants come to the clinic via clinical or self-referral. Within the first three visits, they are assessed using standardized diagnostic interviews, clinical rating scales, and self-report questionnaires. Eligible patients will be asked if they are interested in study participation by clinicians on participating units. Those who are interested will be contacted and formally recruited by the project manager or assistant. They are provided a link to BASS for study consent and the first assessment battery which can be completed directly. Afterwards, they are allocated to either control or intervention group. All participants continue TAU independent of group allocation; for those in the intervention group, LEAP starts when there are enough allocated patients (> 4 patients). After consent, study timeline is at minimum 182 days (three assessment points 90 days apart). Most participants in the intervention group will complete the LEAP program prior to T2 assessment, but due to variation in patient influx at units, some may instead terminate LEAP prior to T3. Participants are no longer included in the study after completed 6-month assessment (T3).

**Fig. 1:**
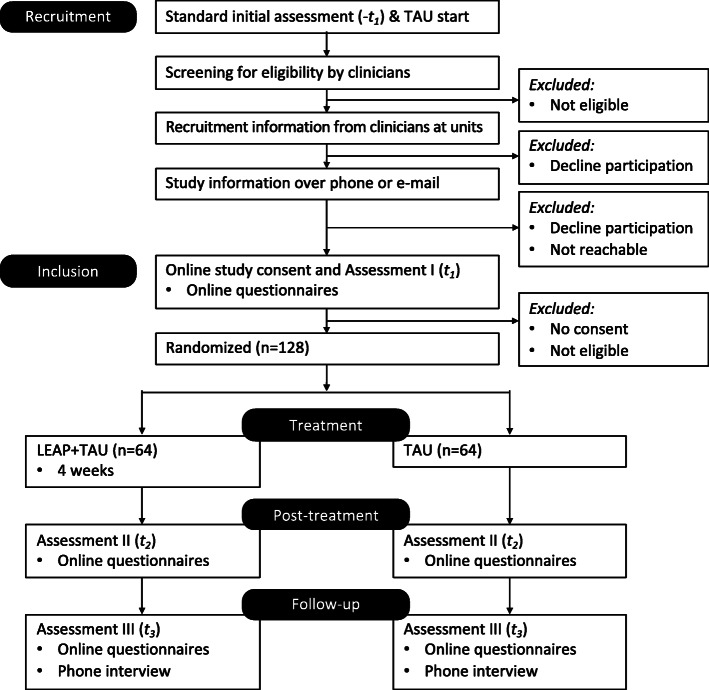
LEAP participant timeline

#### Sample size

Based on pilot data and power analysis with beta = .80, alpha = .05, effect size f = .25 and an estimated correlation between pre- and post-measures of .50, a total of 98 patients are required: 49 patients receiving LEAP and 49 controls. With an estimated drop-out rate of 30%, we will enroll 128 participants (64/arm). As there is a minimum of 4 participants in a LEAP group, length of time between initial assessment and first session of LEAP will vary for patients. This variation will need to be considered a covariate.

#### Recruitment

Patients are recruited at the four participating units. All clinicians at each unit are informed about the project and eligibility criteria and will help identify potential participants; the two LEAP therapists and/or dedicated research managers at the units are formally responsible for recruitment. Eligible patients will be informed about the study; advertisement material is also visible in waiting rooms and from clinicians. To reduce waiting time between recruitment and intervention start, participating units will identify as many potential participants as possible (ideally > 10 patients) prior to asking for participation interest. Upon interest, research contacts at each unit provide contact details to the PI or PC, who will then give oral or written study information to potential participants and provide the link for study consent and first assessment. Non-sensitive personal data (age, gender) for declining participants are collected. Pace of recruitment depends on patient influx on units. One group at a time per unit can be held with a maximum of two groups per semester. With a minimum of four participants per group, each unit could potentially include 16 LEAP participants per year. With four units, the participation goal would then be met within 12 months if equally many control participants are recruited. However, due to covid-19 restrictions, patient influx, and the randomization, a conservative time estimate for inclusion is 24 months (spring 2021 – summer 2023). Status of recruitment is monitored at least monthly with participating units through phone calls and/or e-mails.

#### Allocation

Each eligible patient consenting to participate will be assigned experimental condition using an online random number generator, stratified by site. An allocating investigator (AI) performs the randomization procedure and inform LEAP-therapists and participants. The AI is the only person with access to randomization group data.

#### Blinding

Participants and clinicians cannot be blind to group, since LEAP deviates too much from standard ED treatment. However, the PI will not have access to randomization group data until after the main treatment effects have been analyzed. Most assessments are self-report, but clinicians conducting diagnostic pre- and post-assessments are blinded. The LEAP therapists do not conduct assessments, neither do the PI or PC.

### Data collection and management

#### Outcomes

*Primary outcomes:* remission of ED diagnosis, improved ED psychopathology, and reduced pathological exercise behaviors and cognitions.

*Secondary outcomes:* normalized weight (BMI) and reduced general psychopathology.

#### Instruments/measures

##### Primary outcomes

- Structured Eating Disorder Interview (SEDI [14];) is a semi-structured interview assessing ED diagnostic criteria according to DSM-IV. It is performed at T3; remission status (i.e., ED left or not) is used as outcome (binary data). The SEDI is also performed at three units at initial assessment to determine initial diagnosis. The SEDI has adequate psychometric properties [14]. The Eating Disorder Examination Questionnaire (EDE-Q [15];) is a 28-item questionnaire scored 0–6 (no days-all days) measuring severity of self-reported ED pathology the past 28 days. It generates an overall Global Scale and the subscales Restraint, Eating Concerns, Weight Concerns and Shape Concern. It also measures prevalence of ED behaviors such as restriction, binge-eating, purging and CE. This study will use information of presence and frequency of CE (binary and count data) and ED psychopathology scales as outcomes (continuous data). The EDE-Q has satisfactory psychometric properties [9, 16].
- The Compulsive Exercise Test (CET [17]; is a 24-item questionnaire scored 0–5 (never true-always true) measuring core psychological, behavioral, and emotional aspects of CE. It generates five subscales: Avoidance and rule-driven behavior, Weight control exercise, Mood improvement, Lack of exercise enjoyment, and Exercise rigidity. The LEAP treatment targets the aspects measured by the CET and all subscales are used as outcomes (continuous data). The CET has satisfactory psychometric properties [1, 17, 18].

##### Secondary outcomes

- BMI (weight in kg / [height in m]^2^) is obtained at T1 and T3. At initial registration, some patients are weighed at units (mainly patients with AN), for others, height and weight are self-reported. At T3, BMI is self-reported through the SEDI interview. BMI at T3 is an outcome (continuous data).
- The Difficulties in Emotion Regulation Scale (DERS [19];) short-form (DERS-20) is a 16-item questionnaire rated 1–5 (never-always) measuring emotion dysregulation. The Total score is used in analyses (continuous data). DERS-16 has good psychometric properties [20, 21].
- The DSM-5 Self-Rated Level 1 Cross-Cutting Symptom Measure (DSM-5 symptoms [22]; measures presence and potential severity of general psychopathology in 13 symptom domains. Descriptive data for all domains will be presented and relevant domains may be used in analyses (binary and/or categorical data). The instrument has appropriate psychometric qualities [23].
- Feasibility and acceptance questionnaire, specifically designed for this study, measuring participants satisfaction with structure and content of LEAP (intervention group only; continuous data).

##### Additional measures

- Eating Disorder Examination (EDE [24]) is an interview assessing ED pathology and diagnostic criteria according to DSM-5. One participating unit uses EDE to determine initial diagnosis. The concordance of the SEDI and EDE is high [14]. The EDE has good psychometric properties [25].
- The Active-Q [26] is a questionnaire measuring self-rated physical activity in daily life, both types of and time devoted to the specific activities (categorical and count data). Active Q is a valid and reproducible method for assessing physical activity [26].
- Treatment content questionnaire includes 4 items measuring content of TAU retrospectively and was specifically designed for this study. The questions ask about 1) the main focus of treatment, 2) if exercise has been targeted, if so, estimated percentage of treatment time devoted to exercise, 3) how exercise was targeted (if yes on item 2), and 4) if the respondent has changed his/her approach towards exercise, and if so, how (qualitative data).

#### Data collection methods (Table 2)

##### Data from medical journals

The project uses ED diagnosis from SEDI or EDE, BMI, and the EDE-Q rating as part of T1 data. If project inclusion takes longer than 3 months after initial assessment, these measures are reassessed at the units.

**Table 2:** Schedule of LEAP enrolment, interventions, and assessments (SPIRIT figure)

			STUDY PERIOD			
		Enrolment	Pre-assessment	Allocation	Post-allocation	Close-out
TIMEPOINT	***-t***_***1***_	0	***t***_***1***_		***t***_***2***_	***t***_***3***_
**ENROLMENT:**						
**Eligibility screen**		X				
**Written or oral project information**		X				
**Informed consent**			X			
**Allocation**				X		
**INTERVENTIONS:**						
***LEAP***						
***TAU (both groups)***						
**ASSESSMENTS:**						
***BMI***	X^a^					
***ED diagnosis***	X^a^					
***EDE-Q***	X^a^				X	X
***CET***			X		X	X
***DERS***			X		X	X
***DSM-5***			X		X	X
***Active-Q***			X		X	X
***Treatment content questionnaire***						X
***Feasibility and accept. questionnaire***						X^b^
***SEDI***						X

^a^Part of standard assessment at treatment units at intake

^b^LEAP group only

##### Self-report and interview data

The project includes three assessment timepoints when participants complete questionnaires: T1, 3-month (T2) and 6-month follow-up (T3). Apart from the medical record data at T1, all measurements are distributed to participants through BASS (see below) after study consent. T1 includes self-reports CET, DERS, DSM-5, and Active-Q; T2 includes all T1 self-reports plus the EDE-Q; T3 includes all T2 self-reports plus the treatment content questionnaire (all) and LEAP feasibility and acceptance questionnaire (intervention group only). At T3, the SEDI is also performed by phone.

##### BASS Core Facility

The BASS Core Facility is a secure and encrypted tool for collecting questionnaire data online available through Karolinska Institute. Participants are directed to a secure registration procedure including double authorization with phone and e-mail, and consent to the study through Bank-ID. They also choose their own log-in details which are used subsequently. Each participant is assigned a patient ID used for all further pseudonymization. After study consent, BASS is used for all assessments, BASS manages the timing of later assessments; activation messages to participants and eventual reminders are sent automatically. BASS stores all item level survey data for each participant as soon as one entire instrument is completed; the entire assessment battery does not have to be completed for data to be saved. BASS also stores personal information obtained through the registration procedure (social security number, e-mail, phone number) and study consent, and several types of process data. The contact details are used to send automatic assessment activation messages and reminders. All project related data are stored within BASS for each participant at all timepoints as long as the project is active.

#### Participant retention

Participant retention is promoted by several strategies. Consenting and completing baseline measures through BASS is simple, works on several devices and can be completed at any time of the day. As few measures as possible are used so that assessments consume as little time as possible. Participants assigned to LEAP are given increased attention which is usually regarded as positive. It also begins with an individual session where the intervention is individually conceptualized, assumed to increase personal relevance and motivation. All participants are rewarded with gift cards after completed T2 and T3 assessments. A total of three reminders for late individual assessments are sent automatically every third day, all reminders include a link to BASS. Participants not responding to reminders will then be contacted by project managers. Retention in the LEAP intervention is managed locally at each unit.

#### Data management

*Personal data* (name, contact details) needed for study informing are stored locally at participating units in secure research registries. It is transferred regularly to project managers by phone and entered into a registry. *Medical record data* are transferred to project managers by phone, matched to the correct patient ID (created within BASS upon study consent) and entered into a separate dataset. *Recruitment and intervention data* (non-sensitive personal data (age, gender) for declining participants; intervention related process data) are stored locally at participating units in secure research registries and transferred to project managers after project termination. *Survey, interview, and assessment related data* are stored securely within BASS during the project time; only the project managers and BASS Data Base Administrator (DBA) have access to the database and activity within BASS is logged. After project completion, data will be exported into a separate dataset excluding personal data and deleted in BASS. *Overall project management data* are entered into a research registry at Karolinska Institute. Here, number of participants in each condition is entered as well as information on number of participants, dates, and LEAP therapists for each intervention group at participating units; no personal data are entered here. *Allocation information* including personal data and group is entered into a dataset by the AI and communicated to research contacts at each unit and the participants by phone. All these registries and datasets are stored on a secure server on Karolinska Institute (P-disc under DATA). After study completion, a pseudonymized master datafile will be compiled and stored on the secure KI server (P). A code key matching social security number and patient ID (retrieved from BASS) will be created and stored securely on an encrypted USB-stick in a fireproof safe separate from other data. All other registries containing personal data are destroyed. After project termination, the master datafile will be stored within the KI system for research documentation (ELN) along with the list of variables and related publications. Long-term storage of data follows the KI Archive Act.

#### Confidentiality

All data are kept confidential through the pseudonymized patient ID. During the project time, all project related data that can be connected to individual participants are stored securely at participating units and KI with limited access. The code key matching social security number and patient ID is encrypted and stored securely separate from other data. Analyses will only be performed in pseudonymized datasets.

#### Access to data (who has access to the full dataset)

Only the PI and PC have access to the registries kept during the project time on the secure KI server (P) (except allocation information). After project termination, only the PI and PC has access to the master datafile and code key. Selected data may be shared within the project group trough secure transfer for project related research. The PI determines the appropriateness of such research questions and only permits data access if they are clearly project related and covered by ethical permits.

### Statistical methods

#### Outcomes (methods for analyzing primary and secondary outcomes)

To evaluate the LEAP treatment effect (primary objective) the data will be analysed per protocol and based on intention to treat. The first analysis will include all controls (TAU) and participants that complete the program (LEAP+TAU) and answer the questionnaires. The T3 assessment point will be used for post treatment data unless the participants completed the program before T2. Each continuous outcome measure will be used as a response variable in a linear regression model with group allocation and baseline value as predictors. Logistic regression will be used for categorical outcomes. Additional covariates in the models will include gender, clinic, and time between T1 and first treatment session. Since the removal of drop out and noncompliant subjects in the per protocol analysis may lead to overestimation of the treatment effect, we will also perform the same analysis on the basic of intention to treat. Here all subjects who were randomized to LEAP and answered the questionnaires will be included in the treatment group, independently whether they participated in the LEAP sessions. Assessment points with at least CET and EDE-Q will be included in analyses in relation to the primary outcome. If data is missing on CET and/or EDE-Q, the timepoint will not be considered for analysis. There will be no item level missing data (technically prohibited in BASS). The percentage of patients withdrawing from the program despite the initial intention to participate will be considered as a feasibility marker. To compare proportions of dropout and loss to follow-up within the groups, conventional two-sample Z-tests will be used. To identify prognostic factors associated with a good response to LEAP (secondary objective), the treatment group will be subjected to additional analysis where the outcome of interest is used as response variable in linear or logistic regression model and potential prognostic factors (e.g., BMI, type of ED) are used as predictors. These analyses will include all participants with complete data for each specific model.

### Oversight, monitoring and dissemination

#### Data monitoring

As the intervention has a short duration and minimal known risks, there is no need for a formal data monitoring committee or interim analyses. The principal investigator and project coordinator keep close contact with all participating units and any issues regarding recruitment, intervention, and project overall will be managed continuously. The principal investigator and project coordinator also continuously monitor the project progress and project data (i.e., its completeness and accuracy), and if necessary, consult others in the data management group.

#### Harms

No harms are expected through participation. However, LEAP participants sacrifice time and energy to take part in a treatment program with uncertain benefits while individuals randomized to control group may be disappointed that they will not receive LEAP. Further, although self-rating symptoms as all participants do through BASS is unlikely to be harmful, it may raise concern. Therefore, in all stages of data collection (in visible materials, in BASS, etc.), contact details to the PI are provided, encouraging participants to contact her in case of questions or concerns. Both PI and PC are clinical psychologists and clinicians and/or participants can contact any of them for guidance if needed. As LEAP is delivered in groups, sensitive information might be shared, why the therapists need to make sure that in the first session of every new LEAP group that all participants agree upon a code of conduct. All LEAP therapists are experienced clinicians at the units where participants have an active treatment. Thus, if adverse events arise, participants have access to care and can be monitored. LEAP therapists are also instructed to register any issues related to the LEAP intervention groups, including potential adverse events. This is reported to the PI and managed with appropriate actions.

#### Auditing

No formal auditing is included in the project.

#### Protocol amendments

Modifications in the protocol are communicated to participating units (i.e., LEAP-therapists, research staff; managers, other staff when necessary). Minor modifications may be communicated via e-mail to those concerned, other modifications are communicated through meetings. For significant modifications related to the participants (e.g., eligible criteria, recruitment, assessment, intervention, etc.) or other significant changes, amendments to the ethical permit are applied for. Major modifications (e.g., delays due to covid-19) are also reported to financers and updated in the ISRCT registry.

#### Dissemination policy

Trial results will be published in peer-reviewed psychiatric journals, preferably Open Access to enable wider dissemination. Results will be presented for staff at participating units, to other health-care professionals (e.g., through interest organizations, at research conferences). Results may also be promoted by a public press release and posts on social media. The PI (EFM) is main or senior author, the PC (EM) is co-author on all manuscripts from the project, and the LEAP originator CM is expert advisor. Other authors are based on manuscript contribution. The intervention manual will be available upon demand from the LEAP originator and the PI. Data will not be shared outside of the project group, but meta data will be available upon demand if ethical permits exist (e.g., for meta-analyses).

#### Trial status

Everything is prepared for the study to commence. Collaboration with units is well established, procedures for data collection and recruitment procedures are finalized, and the LEAP training for therapists are completed. However, due to Covid-19 restrictions prohibiting non- essential group interventions at the clinics, recruitment has not been able to start yet. As Covid-19 vaccinations are currently initiated and restrictions possibly eased later on as a result, we plan to start recruiting during the late spring of 2021 with LEAP intervention groups starting as close to recruitment as possible.

## Discussion

The overall project aim is to evaluate the efficacy of the LEAP program targeting CE, modified to suit a population of mixed EDs with CE within an outpatient specialized ED treatment setting. To our knowledge, this is the first study to include patients with other ED diagnoses than AN, when evaluating LEAP. Since CE is a prominent symptom in various ED presentations and is linked to a more negative prognosis, CE requires attention in ED treatment when the patient presents with this problem behavior. LEAP is one of few attempts to address CE systematically and it has the advantage of being a stand-alone, brief treatment delivered as an add-on to standard ED care, thus suitable for almost all patients with CE as a symptom. The present study is naturalistic, which means LEAP will be studied in the setting and form it is intended to work, as opposed to a more controlled scientific context. This is also unique and will provide valuable new information about implementing LEAP in standard ED care.

Currently, about 50% of patients report at least some CE and may therefore benefit from this treatment in terms of overall improved general and ED specific pathology and quicker ED remission. LEAP provide patients with increased knowledge and understanding of the mechanisms that maintain a compulsive stance towards exercise; LEAP also provides tools for promoting healthy and balanced exercise, which is important for relapse prevention. Prior studies have shown that LEAP has positive effects on CE and ED pathology, LEAP also reduced time in inpatient care for individuals with AN. In summary, LEAP has the potential of improving remission, which is of significant importance to afflicted, their families, and the society as a whole in terms of reduced healthcare costs. If LEAP shows beneficial effects in this trial, the goal is to implement LEAP nationally in Sweden. The project group has well-established contacts with the majority of Swedish ED units and as such, is well suited to inform and educate clinicians in the LEAP method. Hopefully, LEAP meets a demand which so far has not been met in specialized ED care. The results of the study will also be disseminated internationally via peer-reviewed journal articles and scientific conferences.

The major trial concerns are related to inclusion pace and LEAP group initiation. As LEAP requires at a minimum 4 participants and because of the randomization procedure, units need to recruit more than twice as many patients at a time to ensure that LEAP groups can start as soon as possible. Recruitment may also be complicated by the RCT-design: potential participants experiencing difficulties related to CE and wanting the intervention may be discouraged from study participation as they may be randomized to the control group. LEAP initiation is not only complicated by participant influx; Covid-19 restrictions has prohibited non-essential group interventions at the clinics and therefore delayed the project. As of now, the hope is that timely vaccination will enable group interventions prior to the summer 2021; in that case, recruitment can start immediately.

### Research ethics approval

The study was approved by the Swedish Ethical Review Agency (number: 2020–01173).

## Data Availability

Not applicable.

## Abbreviations

AN: Anorexia Nervosa
AI: Allocating Investigator
Bank-ID: Bank identification (personal electronic authorization method)
BMI: Body Mass Index
BN: Bulimia Nervosa
CBT-E: Cognitive Behavioral Therapy-Enhanced
CE: Compulsive Exercise
CET: Compulsive Exercise Test
CM: Carline Meyer
DBA: Data Base Administrator
DERS: Difficulties in Emotion Regulation Scale
DSM-IV: Diagnostic and Statistical Manual of Mental Disorders, version 4
DSM-5: Diagnostic and Statistical Manual of Mental Disorders, version 5
ED: Eating Disorder
EDE: Eating Disorder Examination
EDE-Q: Eating Disorder Examination Questionnaire
EFM: Emma Forsén Mantilla
EM: Elin Monell
KI: Karolinska Institute
LEAP: The CompuLsive Exercise Activity TheraPy
OSFED: Other Specified Feeding and Eating Disorders
Patient ID: Patient identification
PC: Project coordinator
PI: Principal Investigator
RCT: Randomized Controlled Trial
SEDI: The Structured Eating Disorder Interview
TAU: Treatment As Usual
TC: Tara Cousins
T1: Timepoint 1 (baseline)
T2: Timepoint 2 (3 months follow-up)
T3: Timepoint 3 (6 months follow-up)
